# Effects of Chaihu-Shugan-San and Shen-Ling-Bai-Zhu-San on p38 MAPK Pathway in Kupffer Cells of Nonalcoholic Steatohepatitis

**DOI:** 10.1155/2014/671013

**Published:** 2014-03-25

**Authors:** Qin-He Yang, Yong-Jian Xu, Yi-Zhen Liu, Yin-Ji Liang, Gao-Fei Feng, Yu-Pei Zhang, Hui-Jie Xing, Hai-Zhen Yan, Yuan-Yuan Li

**Affiliations:** ^1^Medical School of Jinan University, 601 Huangpu Road West, Guangzhou, Guangdong 510632, China; ^2^Experimental Animal Management Center of Jinan University, 601 Huangpu Road West, Guangzhou, Guangdong 510632, China

## Abstract

This study aimed to investigate the effects of Chaihu-Shugan-San (CSS), Shen-Ling-Bai-Zhu-San (SLBZS), and integrated recipe of the above two recipes on inflammatory markers and proteins involved in p38 MAPK pathway in Kupffer cells of NASH rats induced by high fat diet (HFD). Rats were administered at low or high dose of CSS, SLBZS, and integrated recipe except normal group and model group for 16 weeks. The levels of hepatic lipid, TNF-**α**, IL-1, and IL-6 in liver tissues were measured. Kupffer cells were isolated from livers to evaluate expressions of TLR4, p-p38 MAPK, and p38 MAPK by Western blotting. The results showed that the NASH model rats successfully reproduced typical pathogenetic and histopathological features. Levels of hepatic lipid and liver tissues inflammatory factors in high-dose SLBZS group and integrated recipe group were all lower than that of model group decreased observably. Expressions of TLR4, p-p38 MAPK, and p38 MAPK in Kupffer cells were decreased in all treatment groups, but there was no significant difference between treatment groups. The high-dose SLBZS group had the lowest expression levels of TLR4, and the most visible downtrend in the expression levels of p-p38 MAPK and p38 MAPK was found in the high-dose integrated recipe group. The ratio of p-p38 MAPK to total p38 MAPK protein was obviously increased in all treatment groups. Therefore, our study showed that the activation of p38 MAPK pathway in Kupffer cells might be related to the release of inflammatory factors such as TNF-**α**, IL-1, and IL-6 in NASH rats. High dose of SLBZS and integrated recipe might work as a significant anti-inflammatory effect in Kupffer cells of NASH rats induced by HFD through suppression of p38 MAPK pathway. It indicated that p38 MAPK pathway may be the possible effective target for the recipes.

## 1. Introduction

Traditional Chinese medicine (TCM) has been clinically used in China for thousands of years for the treatment of many diseases. Chaihu-Shugan-San (CSS), an ancient classical formula from “Jingyue Quanshu”, is composed of seven Chinese herbs:* Bupleurum Chinese DC*,* Pericarpium Citri Reticulatae*,* Ligusticum chuanxiong Hort*,* Rhizoma Cyperi, Fructus Aurantii*,* Radix Paeonia Alba*, and* Glycyrrhiza uralensis Fisch* with a traditional dose ratio of 6 : 6 : 5 : 5 : 5 : 5 : 3. Shen-Ling-Bai-Zhu-San (SLBZS) is also a famous classical formula recorded in “Taiping Huimin Heji Ju Fang” which consists of ten Chinese herbs:* Panax Ginseng, Atractylodes Ovata, Poria Cocos, Dioscorea Batatas*,* Dolichos lablab*,* Coix lacryma-jobi*,* Nelumbo nucifera*,* Glycyrrhiza uralensis Fisch*,* Platycodon grandiflorum*, and* Amomum xanthioides* in a ratio of 5 : 5 : 5 : 5 : 4 : 3 : 3 : 3 : 2 : 2.

CSS and SLBZS are traditionally used to treat some chronic diseases such as fatty liver disease (FLD) or gastroenteropathy. Many studies have demonstrated that CSS protects against lipid peroxidation [1, 2], liver fibrosis [3, 4], and insulin resistance [5]. And SLBZS has inhibitory activities on oxidative stress [6], lipid peroxidation [7], and inflammatory reaction [6, 8].

Some of the major compounds from CSS and SLBZS, like saikosaponins [9–11], total glucosides of peony [12, 13], ginsenoside [14, 15], atractylenolide [16], atractylodes macrocephalaon polysaccharide [17], and Carboxymethylpachymaran [18], which also have been identified their potential protection on liver. Based on the theory of TCM, CSS dredges liver qi and dispels the stagnation and is prescribed mainly for the liver qi stasis. SLBZS has the functions of tonifying spleen and stomach qi and is mainly used for deficiency of spleen and stomach.

Nonalcoholic steatohepatitis (NASH) is an important stage from simple steatosis development to fibrosis, and cirrhosis in nonalcoholic fatty liver disease (NAFLD), characterized by hepatocellular ballooning degeneration and necroinflammation based on hepatic steatosis [19–21]. Kupffer cells (KCs), which are resident macrophages of the liver, account for 80%–90% of the total innate macrophages [22]. KCs are an important source of both inflammatory and anti-inflammatory mediators [23]. Researches have showed that amounts of inflammatory cytokines and biologically toxic mediators from activated KCs have been strongly implicated in the pathogenesis of hepatic injury, including interleukin-1 (IL-1), interleukin-6 (IL-6), interleukin-10 (IL-10), interleukin-12 (IL-12), interleukin-13 (IL-13), and tumor necrosis factor-alpha (TNF-*α*) [23–25]. Modern researches have also indicated that p38 mitogen-activated protein kinase (p38 MAPK) is closely related to inflammatory cellular signal transduction and gene regulation during the course of NASH [26].

In accordance with our previous study supported by Natural Science Foundation of China (number 30371726), we observed that CSS and SLBZS were significantly effective for the treatment of FLD, respectively [27]. And we found that the high expression levels of phosphor-p38 MAPK (p-p38 MAPK) and p38 MAPK in KCs isolated from 12 weeks high fat diet (HFD)-induced NAFLD rats, which preliminarily revealed the relationship between NAFLD and p38 MAPK pathway [28]. So, how is the NASH rats HFD induced for 16 weeks? In this paper, we studied the effects of soothing liver and invigorating spleen recipe on inflammatory markers and proteins involved in p38 MAPK pathway in KCs of NASH rats induced by HFD in order to explore part of the underlying mechanisms.

## 2. Materials and Methods

### 2.1. Preparation of CSS and SLBZS

CSS is composed of seven Chinese herbs:* Bupleurum Chinese DC*,* Pericarpium Citri Reticulatae*,* Ligusticum chuanxiong Hort*,* Rhizoma Cyperi*,* Fructus Aurantii*,* Radix Paeonia Alba*, and* Glycyrrhiza uralensis Fisch* with a traditional dose ratio of 6 : 6 : 5 : 5 : 5 : 5 : 3. Invigorating spleen recipe includes* Panax Ginseng*,* Atractylodes Ovata*,* Poria Cocos*,* Dioscorea Batatas*,* Dolichos lablab*,* Coix lacryma-jobi*,* Nelumbo nucifera*,* Glycyrrhiza uralensis Fisch*,* Platycodon grandiflorum*, and* Amomum xanthioides *in a ratio of 5 : 5 : 5 : 5 : 4 : 3 : 3 : 3 : 2 : 2. Integrated recipe contains is the mixture of CSGS and SLBZS at a ratio of 1 : 1. All Chinese medicines were formula granules purchased from Shenzhen Sanjiu Medical Co., Ltd. (1005001S). The formula granules were put in the solvent of distilled water and preserved at −4°C refrigerator.

### 2.2. Animals, Grouping, and Modeling

120 Specific Pathogen-Free Male Sprague-Dawley rats (6 weeks old, 200 g ± 20 g) were obtained from the Laboratory Animal Research Center of Guangzhou University of Traditional Chinese Medicine (Approval number SCXK (Yue) 2008-0020), Guangdong province, China. The rats were housed under conditions of controlled temperature (24°C ± 2°C) and humidity (70% ± 10%) in 12 h of light and 12 h of dark cycle (lights on from 8:00 am to 8:00 pm), with free access to diet and water. After one week of adaptive breeding, the rats were randomly divided into 8 groups, 15 rats in each group: normal group, model group, low-dose CSS group (L-CG), high-dose CSS group (H-CG), low-dose SLBZS group (L-SG), high-dose SLBZS group (H-SG), low-dose integrated recipe group (L-IG), and high-dose integrated recipe group (H-IG). Rat models of NASH were duplicated according to method as we previously reported [29] with some minor modifications. Normal group of rats got free access to a normal chow diet, model group of rats were fed with HFD (composed of regular chow 88%, axungia porci 10%, cholesterol 1.5%, and bile salt 0.5%). All rats in treatment groups were fed with decoction (1 mL/100 g body weight by gastrogavage [30]), while the rats in the normal group and model group were fed with the same dose of distilled water once at 8:00 am every day. Low-dose equaled human clinical equivalent dosage, and high-dose was 3-fold volume of low-dose. The treatment lasted for 16 weeks.

At the end of treatment, rats in each group were divided into two groups by table of random number: 9 rats for liver samples collection, 6 rats for isolation of KCs. All rats were treated in compliance with the Guiding Principles for Animal Experiments and the protocols were approved by the Animal Experimental Ethnics Committee of Jinan University, China.

### 2.3. Biochemical Test in Liver

After rats were anesthetized by intraperitoneal injection of 3% pentobarbital (0.2 mL/100 g body weight), livers were taken out quickly. Liver tissues were put into isopropanol. Homogenates were manufactured using a TissueLyser-II homogenizer (QIAGEN, Germany), centrifuged at 3000 ×g, 4°C for 10 min, and then clear supernatants were collected. Total cholesterol (TC) and triglyceride (TG) in the liver tissue were determined with automatic biochemical analyzer (Olympus, Japan).

### 2.4. Histopathological Examination of Liver

The paraffin-embedded liver tissue (about 1 cm × 0.5 cm × 0.5 cm) which selected the same part of the liver, about 0.5 cm from the edge of the right hepatic lobule, was sliced at a thickness of 4 *μ*m and examined by hematoxylin-eosin (HE) staining. The steatosis grade, fibrosis stage, and inflammation of NASH were evaluated according to the NASH histological scoring system [31].

### 2.5. Determination of Inflammatory Cytokines in Liver Tissue

Liver homogenates were centrifuged at 3000 ×g, 4°C for 10 min. Clear supernatants were used to determine the cytokines. The contents of TNF-*α*, IL-1, and IL-6 were tested following the recommended procedures provided by the enzyme-linked immunosorbent assay (ELISA) kits.

### 2.6. Separation and Identification of KCs

KCs were isolated and identified from 6 rats in each group as we previously described [32], and some modifications were made. After rats were anesthetized, the liver was perfused in situ with 200 mL 0.5 mmol/L Ethylene Glycol Tetraacetic Acid (EGTA) in D-Hanks at 20 mL/min, 37°C until the colour of liver changed into amber. Then the liver was transferred to a culture dish and was perfused ex situ with 0.03% collagenase IV in Hanks, which contains 5 mmol/L calcium ion and should be preheated to 37°C, at 20 mL/min in a recirculating fashion for 15 min. The liver was then placed into 10 mL RPMI-1640 culture medium containing 10% fetal calf serum (FBS), capsule and fibrous tissue were removed, and the remaining tissue was cut into small pieces. After the obtained liver homogenate was filtered through 200 *μ*m and 300 *μ*m nylon mesh, the cell suspension was centrifuged at 50 ×g, 4°C for 3 min and clear supernatant was collected in another tube and centrifuged at 400 ×g, 4°C for 10 min. The cell pellet was subsequently resuspended in RPMI-1640 containing 10% FBS.

Then some 15 mL centrifuge tubes were carefully laid into 2.5 mL 24% Nycodez working solution in the bottom, 2.5 mL 11% Nycodez working solution in the middle layer, and 2.5 mL the cell suspension in the top. Then it was centrifuged at 800 ×g, 4°C for 15 min. KCs which have a clouding appearance between 11% Nycodez layer and 24% Nycodez layer were collected to another 15 mL tube and resuspended in GBSS, and then centrifuged at 400 ×g, 4°C for 15 min twice. The cell pellet was then resuspended and seeded on culture dish at a density of 2–5 × 10^6^ cells/mL with RPMI-1640 containing 10% FBS and incubated in a 5% CO_2_ atmosphere for 30 min at 37°C. By further using adhesion purification, KCs purity was improved, and cell viability was tested by trypan blue dye exclusion.

### 2.7. Protein Extraction and Western Blot

Western blotting was used to determine proteins of KCs toll like receptor 4 (TLR4), p-p38 MAPK, p38 MAPK, and Glyceraldehyde-3-phosphate dehydrogenase (GAPDH). GAPDH was used as an internal control. KCs were split in RIPA lysis buffer and centrifuged at 8000 ×g for 5 min at 4°C and the supernatants were collected. The supernatant protein concentration was determined by BCA protein assay. Sixty micrograms of protein was resolved by 10% sodium dodecyl sulfate polyacrylamide gel electrophoresis (SDS-PAGE) and proceeded with transmembrane. The polyvinylidene difluoride (PVDF) membrane was blocked with 5% skim milk in Tris-Buffered Saline Tween-20 (TBST), shaken for 1 h at room temperature, and then incubated overnight at 4°C with specific primary antibodies. Then horseradish peroxidase (HRP) conjugated goat-anti-rabbit antibody were added and incubated at room temperature for 1 h. After being washed three times in TBST, the PVDF membrane was put into developer and exposed to X-ray film. The films were scanned and analyzed by gel image processing system.

### 2.8. Statistical Analysis

The results were expressed as the mean ± S.E.M. unless otherwise indicated. Analysis of variance (ANOVA) was used to determine the statistical significance of the differences followed by Tukey's test. Ranked data were analyzed by Rank-Sum test. Probability value (*P*) less than 0.05 was considered statistically significant. All data were analyzed with the Statistical Package for the Social Sciences (SPSS, USA) 13.0 Software.

## 3. Results

### 3.1. Levels of TC and TG in Liver

**Figure 1:**
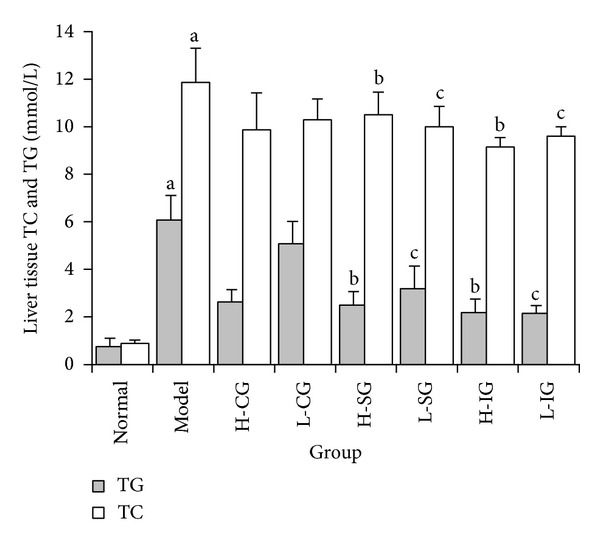
Levels of TC and TG in liver were determined. Rats were fed with normal chow diet or HFD with or without CSS and SLBZS for 16 weeks. The values were expressed as mean ± S.E.M. of 9 rats per group. ^a^ *P* < 0.01 versus normal group; ^b^ *P* < 0.01, ^c^ *P* < 0.05 versus model group.

Elevated levels of TC and TG indicated hepatic lipid accumulation and lipid metabolic disturbance in liver tissue. As shown in Figure 1, the levels of TC and TG were significantly increased in the model group compared to the normal group (*P* < 0.01). Compared with the model group, lower levels of TG and TC were shown in the H-SG, L-SG, H-IG, and L-IG (*P* < 0.01, *P* < 0.05). Results indicated the increased TG and TC induced by HFD were attenuated by high and low dose of SLBZS and integrated recipe.

### 3.2. Effects of CSS and SLBZS on Liver Histopathological Changes

**Figure 2:**
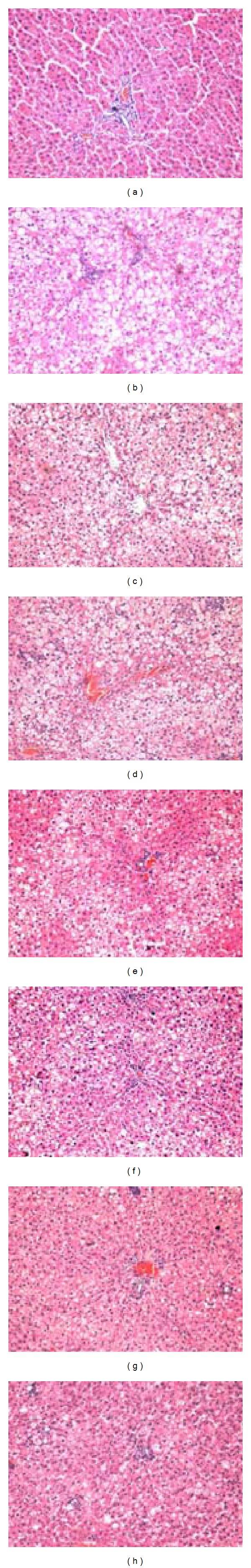
Histological changes of liver sections in different groups (HE stain × 100). (a): normal group; (b): model group; (c): high-dose CSS group (H-CG); (d): low-dose CSS group (L-CG); (e): high-dose SLBZS group (H-SG); (f): low-dose SLBZS group (L-SG); (g): high-dose integrated recipe group (H-IG); (h): low-dose integrated recipe group (L-IG).

**Figure 3:**
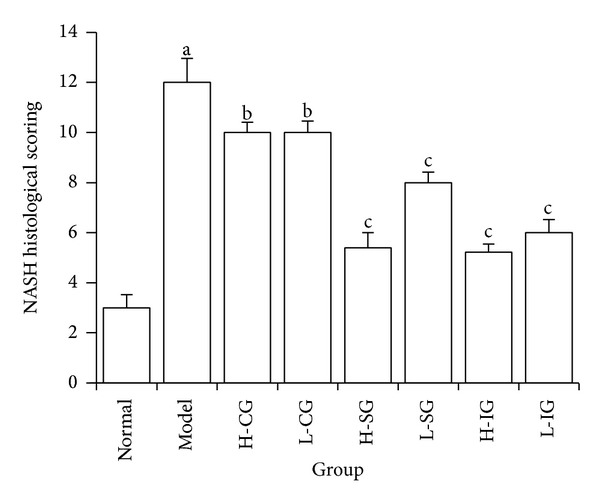
NASH histological scoring in different groups. Rats were fed with normal chow diet or HFD with or without CSS and SLBZSs for 16 weeks. The values were expressed as mean ± S.E.M. of 9 rats per group. ^a^ *P* < 0.01 versus normal group; ^b^ *P* > 0.05, ^c^ *P* < 0.01 versus model group.

Liver specimens with HE staining were shown in Figure 2. Sections of liver from model group showed typical NASH features, including microvesicular and macrovesicular steatosis, lobular and portal inflammation, fibrosis, and hepatocyte ballooning (Figure 2(b)). Compared with the normal group, the model group scored 12 points and had a significant difference (*P* < 0.01). The pathological changes in the treatment groups lightened to different degree as compared with the model group, particularly in H-SG, L-SG, H-IG, and L-IG (*P* < 0.01) (Figure 3). This indicated that the liver steatosis, fibrosis, and inflammation were inhibited to some extent by both high and low dose of SLBZS and integrated recipe in NASH rats.

### 3.3. Effects of CSS and SLBZS on Liver Inflammatory Cytokine Levels

**Figure 4:**
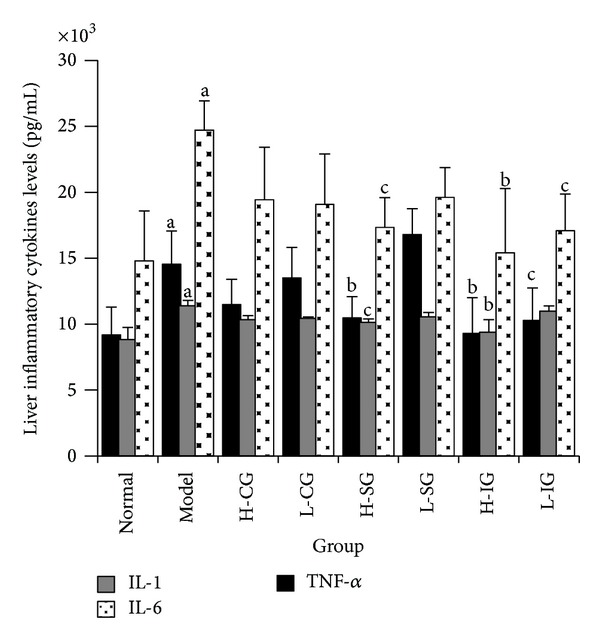
Related inflammatory cytokines of TNF-*α*, IL-1, and IL-6 in liver tissues were determined by ELISA. Rats were fed with normal chow diet or HFD with or without CSS and SLBZSs for 16 weeks. The values were expressed as mean ± S.E.M. of 9 rats per group. ^a^ *P* < 0.01 versus normal group; ^b^ *P* < 0.01, ^c^ *P* < 0.05 versus model group.

Rising inflammatory cytokine levels of TNF-*α*, IL-1, and IL-6 are regarded as biomarkers of inflammation. As shown in Figure 4, higher levels of TNF-*α*, IL-1, and IL-6 were observed in the model group compared with that of the normal group (*P* < 0.01). Compared with the model group, significant decreases of TNF-*α* and IL-6 in the H-SG, H-IG, and L-IG (*P* < 0.01 or *P* < 0.05), and the levels of IL-1in H-SG and H-IG were clearly lower (*P* < 0.01 or *P* < 0.05). The results showed that both the high dose of SLBZS and integrated recipe reduced the TNF-*α*, IL-1 and IL-6 levels of liver inflammatory cytokine in NASH rats induced by HFD.

### 3.4. The Population, Purity, and Viability of KCs

The yields of purified cell of KCs in each rat were 1.5–2.0 × 10^7^. The viability of KCs isolated was higher than 95%, with purity over 90.18%. The number and purity degrees of KCs complied with the requirement of the follow-up testing.

### 3.5. Effects of CSS and SLBZS on p38 MAPK Signal Pathway Related Proteins in KCs

**Figure 5:**
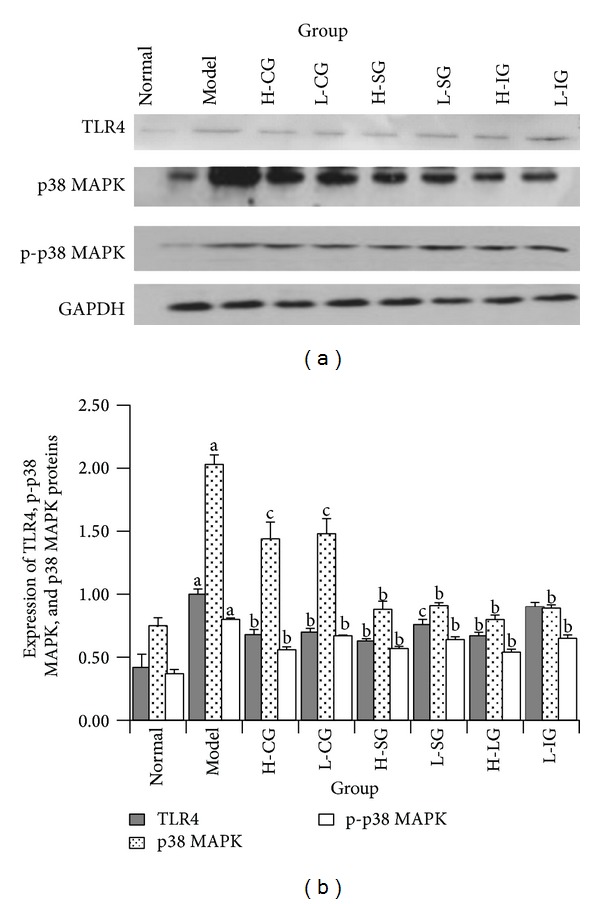
Western blot analysis of proteins involved TLR4, p-p38 MAPK, and p38 MAPK in Kupffer cells (a). Expression of TLR4, p-p38 MAPK, and p38 MAPK proteins in Kupffer cells (b). Rats were fed with normal chow diet or HFD with or without CSS and SLBZSs for 16 weeks. KCs were isolated and identified from 6 rats in each group. Values represent the mean ± S.E.M. ^a^ *P* < 0.01 versus normal group; ^b^ *P* < 0.01, ^c^ *P* < 0.05 versus model group.

**Figure 6:**
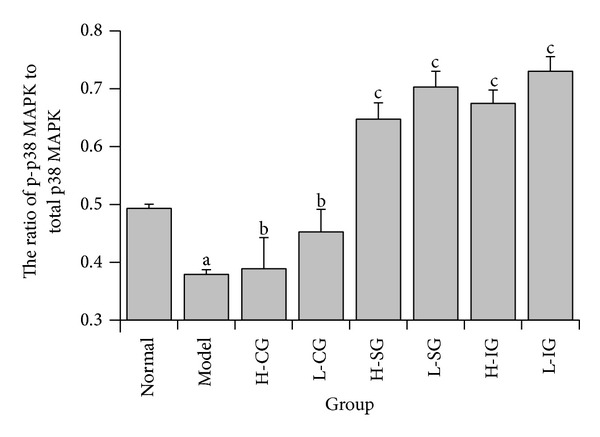
The ratio of p-p38 MAPK to total p38 MAPK protein in Kupffer cells. Rats were fed with normal chow diet or HFD with or without CSS and SLBZSs for 16 weeks. KCs were isolated and identified from 6 rats in each group. Values represent the mean ± S.E.M. ^a^ *P* < 0.05 versus normal group; ^b^ *P* > 0.05, ^c^ *P* < 0.01 versus model group.

To explore the mechanism of the anti-inflammatory effect of soothing liver and SLBZS in KCs of NASH rats, we assayed three important proteins of TLR4, p-p38 MAPK, and p38 MAPK involved in p38 MAPK signal pathway which is one important mediator in inflammatory response. Figures 5(a) and 5(b) showed that protein expression levels of TLR4, p-p38 MAPK, and p38 MAPK in the model group were significantly higher than those in the normal control group (*P* < 0.01). Compared with the model group, the expression levels of TLR4, p-p38 MAPK, and p38 MAPK (*P* < 0.01, *P* < 0.05) were inhibited in all treatment groups, but there was no significant difference between treatment groups. The group of H-ISG had the lowest expression levels of TLR4, and the most visible downtrend in the expression levels of p-p38 MAPK and p38 MAPK was found in the group of H-IG. Compared with the model group, Figure 6 showed that the ratio of p-p38 MAPK to total p38 MAPK protein increased obviously (*P* < 0.01), but H-CG and L-CG did not (*P* > 0.05). The result indicated that p38 MAPK signal pathway may be activated in KCs of NASH rats. The high dose of SLBZS and integrated recipe inhibited activation of p38 MAPK signal pathway in different degrees.

## 4. Discussion

NASH is a common chronic liver disease, and it has been one of the important factors in leading to hepatocirrhosis and liver cancer [33, 34]. It turns out that the excessive inflammatory cytokines such as TNF-*α* [35–38], IL-1 [36, 39], and/or IL-6 [36, 40–42] exacerbated cell lipid peroxidation and liver injury and promoted NASH progression in different ways.

On present understanding, MAPKs are a highly conserved family of serine/threonine kinases including known ERK 1/2, JNK/SAPK, p38 MAPK, and ERK5/BMK1, which are all important signaling molecules in the control of cellular biological effects to extracellular stimuli. Following stimulation, the proteins of p38 MAPK signal pathway are phosphorylated and then activate several downstream factors to regulate the corresponding gene expression [43]. And the study of Wagner EF showed that p38 MAPK signal pathway played an important role in the stress responses of inflammatory reaction [44]. TLR4 is the main receptor in the lipopolysaccharide- (LPS-)mediate immune responses [45]. After TLR4 is integrated with LPS, MAPKs cascade reactions are activated by the pathway of myeloid differentiation factor (MyD88), interleukin-1 receptor related kinase-1 (IRAK-1), tumour necrosis factor receptor correlation factor (TRAF6), and transforming factor activating kinase (TAK1). Then the p38 MAPK protein is phosphorylated, leading to release of inflammatory factor and starting cell damage mechanism [46, 47]. Moreover, the activated TLR4 pathway turned out to be playing a critical role in the inflammatory immune response of NASH [48] and it was demonstrated that nosogenesis of many inflammatory diseases was mediated with TLR4-p38 MAPK signal pathway [49, 50].

In the present research, rat model of NASH induced by HFD successfully replicated several typical histopathological characteristics of NASH in human, such as hepatocyte steatosis and ballooning and lobular and portal inflammation. And levels of TC, TG, and inflammatory factors in liver were increased in different degrees. It was consistent with the previous reporter [29]. Our preliminary studies have suggested that CSS, SLBZS, and integrated recipe have certain therapeutic effect on FLD [27, 28, 51] and NFLD [52]. In this research, the results showed that the high and low dose of SLBZS and integrated recipe protected against liver injury, moderated NASH progression, and decreased liver lipid and inflammatory factors levels.

To elucidate how CSS and SLBZS affect p38 MAPK signal pathway and the anti-inflammatory, we detected several proteins which were closely related to the signal transduction of p38 MAPK pathway in KCs of NASH rats. The results demonstrated that the activation of TLR4-p38 MAPK signal pathway in KCs was involved in the development of NASH induced by HFD. The increases of TNF-*α*, IL-1, and IL-6 might be due to the activation of TLR4-p38 MAPK signal pathway in KCs. Both the high dose of SLBZS and integrated recipe may inhibit the related proteins expression in TLR4-p38 MAPK signal pathway to decrease inflammatory factors such as TNF-*α*, IL-1, and IL-6. Moreover, it was interesting to note that the high and low dose of CSS inhibited activation of TLR4, p-p38 MAPK, and p38 MAPK in different degrees, but there was no significant difference compared with the model group on the contents of TNF-*α*, IL-1, and IL-6 and the ratio of p-p38 MAPK to total p38 MAPK protein. So we did not observe that the phosphorylation of p38 MAPK was suppressed by the high and low dose of CSS.

Based on the theory of TCM, CSS dredges liver qi and dispel the stagnation and is prescribed mainly for the liver qi stasis. SLBZS has the functions of tonifying spleen qi and is mainly used for deficiency of spleen and stomach. In accordance with the previous study, we suggested that the basic pathogenesis of FLD was closely correlated to liver stagnation and spleen deficiency from the point of TCM theory [27, 28, 51, 53, 54]. In this study, the effects of high-dose SLBZS and high-dose integrated recipe are better than that of high- or low-dose CSS. Thus we suggested that the pathogenesis of NASH might be closely related to Pixu in NASH rats induced by HFD for 16 weeks.

## 5. Conclusion

In conclusion, this study revealed that the activation of p38 MAPK pathway in Kupffer cells might be related to the release of inflammatory factors such as TNF-*α*, IL-1, and IL-6 in NASH rats. High dose of SLBZS and integrated recipe might work as a significant anti-inflammatory effect in Kupffer cells on NASH induced by HFD through suppression of p38 MAPK pathway. At the same time, p38 MAPK pathway may be the effective targets for the recipes. Thus, SLBZS and integrated recipe might be a potentially complementary medicine used in the treatment of NASH.

The Chinese medicinal herbs exert their pharmacological effects usually through a multicomponent and multitarget way. Further study is needed to find out whether there is some other signal transduction pathways involved in the course and to elucidate the other beneficial effect of CSS and SLBZS on NASH. Moreover, it is essential to do some evidence-based medical research on CSS and SLBZS in clinical applications.
